# SyntTax: a web server linking synteny to prokaryotic taxonomy

**DOI:** 10.1186/1471-2105-14-4

**Published:** 2013-01-16

**Authors:** Jacques Oberto

**Affiliations:** 1 Université Paris-Sud 11, CNRS, UMR8621, Institut de Génétique et Microbiologie, 91405, Orsay, France

## Abstract

**Background:**

The study of the conservation of gene order or synteny constitutes a powerful methodology to assess the orthology of genomic regions and to predict functional relationships between genes. The exponential growth of microbial genomic databases is expected to improve synteny predictions significantly. Paradoxically, this genomic data plethora, without information on organisms relatedness, could impair the performance of synteny analysis programs.

**Results:**

In this work, I present SyntTax, a synteny web service designed to take full advantage of the large amount or archaeal and bacterial genomes by linking them through taxonomic relationships. SyntTax incorporates a full hierarchical taxonomic tree allowing intuitive access to all completely sequenced prokaryotes. Single or multiple organisms can be chosen on the basis of their lineage by selecting the corresponding rank nodes in the tree. The synteny methodology is built upon our previously described Absynte algorithm with several additional improvements.

**Conclusions:**

SyntTax aims to produce robust syntenies by providing prompt access to the taxonomic relationships connecting all completely sequenced microbial genomes. The reduction in redundancy offered by lineage selection presents the benefit of increasing accuracy while reducing computation time. This web tool was used to resolve successfully several conserved complex gene clusters described in the literature. In addition, particular features of SyntTax permit the confirmation of the involvement of the four components constituting the *E. coli* YgjD multiprotein complex responsible for tRNA modification. By analyzing the clustering evolution of alternative gene fusions, new proteins potentially interacting with this complex could be proposed. The web service is available at http://archaea.u-psud.fr/SyntTax.

## Background

The conservation of gene order or synteny has become an invaluable method for establishing the orthology of genomic regions in different species and to infer functional relationships between genes. The term synteny ('same ribbon' in Greek) was introduced four decades ago to define loci positioned on the same chromosome whether they are genetically linked or not [1]. Synteny has since notably diverged from the original definition and commonly refers to gene loci in different organisms located on a chromosomal region of common evolutionary ancestry [2]. For simplicity, the term synteny will be used hereafter to indicate conservation of gene order even if some would prefer the more accurate 'shared synteny' denomination. In the last years, large scale sequencing has increased the number of complete prokaryotic genomes exponentially and endows synteny with an even more prominent role. This wealth of genomic information has motivated the development of new bioinformatics tools capable of processing large amounts of data in order to produce valid synteny analyses. A number of algorithms and related implementations have been developed for the automatic identification of syntenies across multiple genomes (see [3] for a review). If these algorithms are able to predict all conservations of gene order at the genome level, they require very intensive computations which restricts their use to a finite group of organisms. These tools are therefore not adapted for the retrieval of a particular gene synteny in a set of newly sequenced genomes. Such queries, commonly performed by geneticists and phylogeneticists require tools of a different nature, able to provide promptly up to date syntenies in human-readable form. Several web services have been developed for this purpose such as GeConT2 [4], PSAT [5] and GCView [6]. Unfortunately, syntenies produced by these tools are pre-calculated and therefore often outdated. Their relevance depends on the variable frequency of their genome updates. Standalone synteny programs such as GeneclusterViz [7] are also available but require in addition to local installation, the impractical retrieval, manipulation and storage of large data files. To address most of these limitations, we recently developed the Absynte web server [8].

The large and continuously increasing number of completely sequenced prokaryotes is introducing yet another level of difficulty. Researchers working on gene syntenies in given species might overlook their relationships with newly sequenced organisms and potentially miss relevant conserved gene clusters. Indeed, with over 2000 sequenced micro-organisms often responding to exotic genus names, keeping a mental track of all the lineages is a daunting task. To my knowledge, with the exception of GeCont2 [4] which offers partial access to phyla, none of the afore mentioned synteny tools allows organism selection on the basis of taxonomy. The SyntTax web service described here aims to assist the analysis of synteny by providing an organized tree exposing the full lineage of every completely sequenced prokaryote in the National Center for Biotechnology Information (NCBI) repository. In this manner, single or multiple organisms can be easily selected by any combination of classification ranks allowing the generation of robust syntenies performed according to taxonomic criteria. The taxonomic and genomic databases are stored locally on the SyntTax server; they are both updated automatically on a daily basis from the respective NCBI resources. This feature makes of SyntTax a flexible tool capable of promptly adapting to the addition of new sequenced genomes. The validity of the SyntTax web service was tested in benchmarking experiments based on conserved complex gene clusters described in the literature. Additionally, a new use for SyntTax syntenies was also investigated in the prediction of additional functions for the YgjD multiprotein complex by analyzing the taxonomic distribution of gene fusions.

## Implementation

### Features

**Figure 1:**
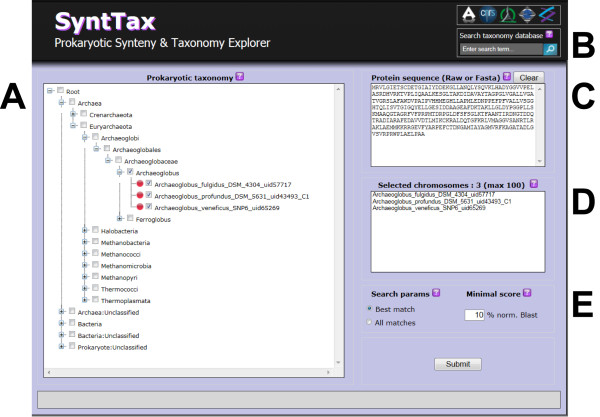
**SyntTax interface.** (**A**) Taxonomic tree allowing the selection of single or multiple genomes on the basis of their lineage. (**B**) The taxonomy database can be searched for single terms in order to retrieve the corresponding complete lineages. (**C**) A user-defined protein sequence can be introduced in any format including FASTA; numerals, blank spaces, and carriage returns are removed automatically. (**D**) Up to 100 individual genomes can be selected from the taxonomic tree and are listed for clarity. (**E**) The genomic BLAST search parameters can be user selected in order to allow all hits or to limit the search to the best hit for each genome. The degree of orthology can be user-selected between 10 and 100%.

**Figure 2:**
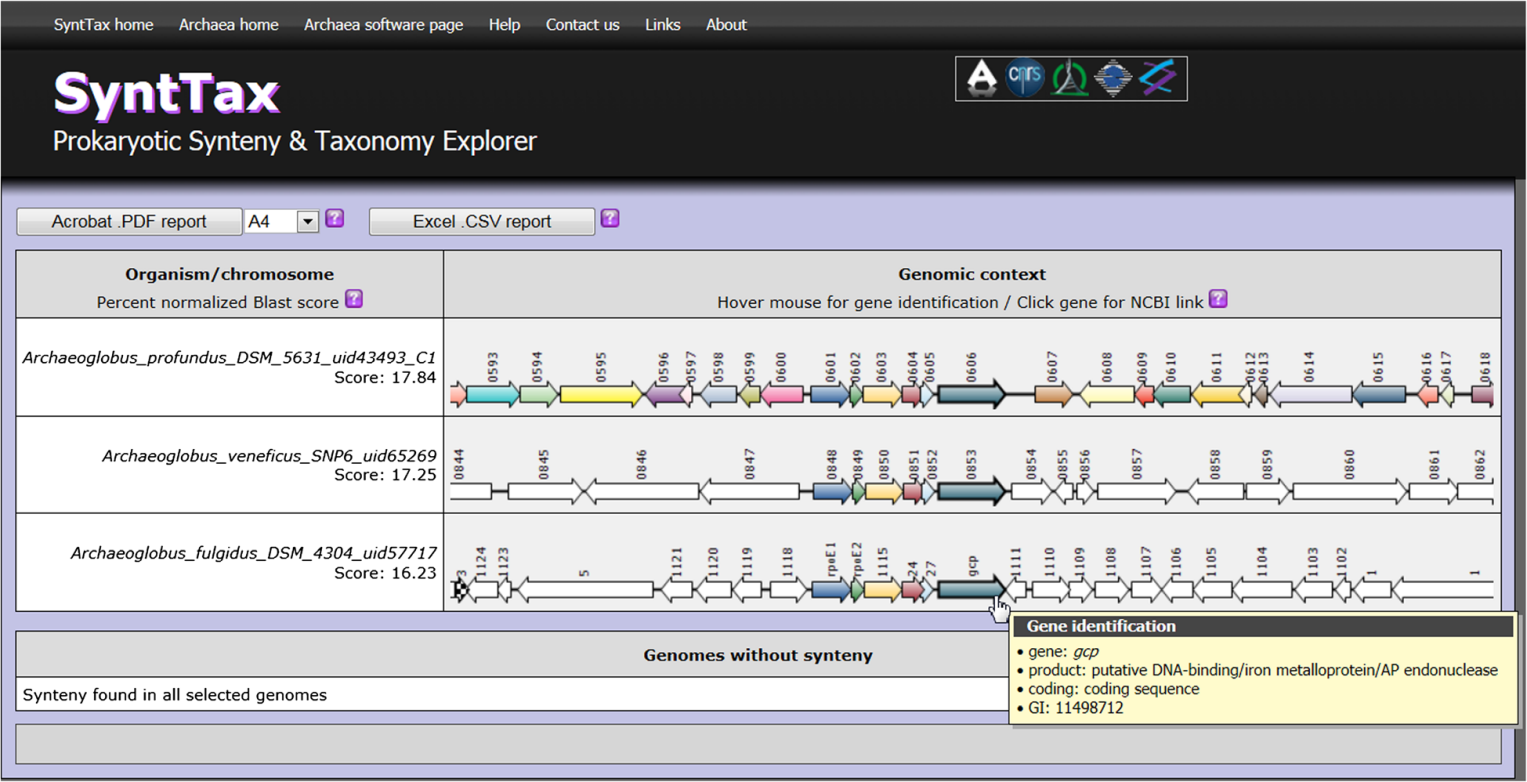
**SyntTax result page example.** The Kae1 synteny obtained for the three available *Archaeoglobus* genomes shows a rapid divergence outside of a small cluster containing five genes upstream Kae1. Specific additional information is available for each gene in the synteny by mouse hovering. Outside links to NCBI protein definitions are also available by clicking on the corresponding gene symbol. SyntTax reports can be exported in Excel 'csv' or high definition Acrobat 'pdf' formats for storage or further elaboration.

The SyntTax interface was designed to be user-friendly and straightforward (Figure 1). Up to 100 individual genomes can be selected (Figure 1D) from the taxonomical tree (Figure 1A). The genomes selected in this manner are then matched against a user-defined protein sequence (Figure 1C). Upon user decision, the synteny can be either limited to the best hit for each genome or include all hits (Figure 1E). The minimal normalized genomic BLAST scores can be user-specified to control the degree of orthology as described by Lerat *et al*. [9]. The complete taxonomy database can be searched to retrieve complete lineages matching the query term (Figure 1B). If the SyntTax search is successful, the resulting synteny is displayed as an interactive graphical table (Figure 2). The consistent color code used for the genetic maps allows the immediate perception of gene clustering. Since the gene labels are rarely explicit, contextual tooltips displaying salient GenBank annotation are available for each gene by mouse hovering. Direct access to the NCBI protein sequence repository is also provided by clicking on the respective gene symbol. The results page displays at the bottom the list of selected genomes where the synteny was not found. Syntax reports can be exported either as a high-resolution vector-oriented graphics in Acrobat 'pdf' or in detailed Excel 'csv' formats for storage or further processing. Additional contextual information under the form of help icons is available throughout the web service interface and results page.

### Retrieval of the taxonomic data

The main resource for lineage information is provided by the NCBI Taxonomy database [10]. The genus and species definers for each organism are obtained during the update of the corresponding genome from the NCBI repository (ftp://ftp.ncbi.nih.gov/genbank/genomes/Bacteria). The taxonomy data originates from the same source and is accessed and retrieved using the Entrez Programming Utilities (E-utilities) [11] publicly available at http://eutils.ncbi.nlm.nih.gov/entrez/eutils/. A first 'esearch' request permits the retrieval of the lineage of a particular organism using its genus and species name italicized in the following command:

$$\begin{array}{c} \text{esearch}.\text{fcgi}?\text{db}=\text{taxonomy}\&\text{term}\\ \;=\left[\text{genus}\;\text{species}\right]\left[\text{LNGE}\right] \end{array}$$

The response consist of a list of identification numbers describing the complete taxonomy of the organism. A subsequent 'esummary' request using this list of *id*_*0*_*.id*_*n *_identifiers retrieves the corresponding taxonomical definitions and ranks using the command below:

$$\text{esummary}.\text{fcgi}?\text{db}=\text{taxonomy}\&\text{id}=\left[id_{0}.id_{n}\right]$$

This procedure allows obtaining, for each organism, the superkingdom, phylum, class, family and order definitions in addition to the already available genus and species definitions present in the FTP directory.

### Taxon processing

In order to cover all the organisms included in the NCBI database, particular attention was devoted to maximize the retrieval of taxonomic data. Most lineages originating from the NCBI are complete, displaying a constant rank depth of 7 with all ranks defined. However, a number of organisms labeled 'candidatus' or 'uncultured' often lack one or more rank definitions. The addition to the database of incompletely defined lineages without proper processing would impair the uniqueness of particular ranks and perturb the subsequent recursive addition of new taxa. When at least the superkingdom is defined, the incomplete taxa are shortened to superkindom and phylum definitions (if the latest is available) and added under Bacteria:unclassified or Archaea:unclassified. Severely incomplete lineages are stored under Prokaryote:unclassified.

### Storage and update of the taxonomic database

On the SyntTax web server, the taxonomical information is stored as an XML document whose hierarchical structure is particularly well suited to store data of this nature. The SyntTax taxonomy database is fully accessible to the user and can be queried for particular organism names. New routines devoted to the management of the taxonomy database have been added to the ancillary Updater program described previously [8,12,13]. This program is now capable of performing automated daily incremental updates of both the taxonomic and genomic databases. Decremental updates are performed as well in order to remove obsolete, redundant or renamed organisms from the local databases.

### Improvement of the Absynte synteny algorithm

The synteny methodology used in SyntTax is based on the Absynte algorithm described in our previous work [8] with significant improvements. The algorithm employs a multiple center star gene clustering topology and can be briefly summarized as follows. The query protein is first compared to itself using BLASTP [14] in order to permit normalization of further alignments. The same protein sequence is then compared to the DNA sequence of the selected target genomes using TBLASTN [14]. The normalized results obtained in such queries allow ranking of the various genomes by decreasing scores and to extract the absolute chromosomal coordinates of the matching hit. DNA segments of 15kb, centered on these coordinates are then extracted from each of these chromosomes and translated according to corresponding GenBank annotation. The proteins of the highest ranking chromosome are compared to each other using the Smith-Waterman-Gotoh (SWG) global alignment algorithm for performance reasons detailed in the Results and Discussion section. A unique color is assigned to each individual protein or to paralogs, when present. These protein sequences are then compared to those extracted from the remaining chromosomes using SWG and the newly identified orthologs are colored accordingly. The synteny becomes readily apparent upon proper alignment and proportional drawing of the color-coded genetic maps. All the processing mentioned above is executed in real time and does not rely on pre-calculation. Synteny analysis with the Absynte/SyntTax algorithm is therefore a processor-intensive task and a number of measures were taken in order to ensure optimal performance. Faster volatile memory access was favored over slower disk operations. Disk I/O was limited to database reading as no user data is written on the server at any time. Multi-threaded or parallel operations were also developed in most areas of the algorithm in order to take full advantage of the multi-core processors architectures available in modern servers. A specific area of the synteny algorithm, build around the BLAST executable (v2.2.24) was nevertheless still single-threaded in the original Absynte algorithm. A substantial optimization of the algorithm could be achieved in SyntTax by allowing full multi-threaded execution of BLAST v2.2.26 in addition to the already parallel SWG routines. The entire data flow and processing can now be distributed among the individual processors. The result report produced by SyntTax was also significantly improved: a list of the selected genomes where the synteny is not observed is now included in the result page and in the Acrobat .pdf and Excel .csv reports. In addition, the sequence of the query protein is also included in the printable reports.

## Results and discussion

Synteny analysis in real time is a processor-intensive task. To address this problem, SyntTax permits the selection of target organims on the basis of taxonomy in order to reduce computing time and increase the robustness of the analysis. With the relatedness of organisms becoming readily apparent, the user is able to restrict the search to more meaningful organisms while avoiding the redundancy of too closely related species. Several benchmarking experiments were performed in order to demonstrate the capabilities of the SyntTax web service and to evaluate its efficiency. In the first part of this section, SyntTax was tested for the resolution of two conserved complex gene clusters reported in the literature. In the last part, I will describe a predictive analysis aiming to determine new potential factors interacting with the *E. coli* YgjD complex recently described as involved in tRNA modification [15]. This new use for synteny is directly correlated to the specific taxonomic capabilities of SyntTax and illustrates the potential of this new web service.

### Performance considerations

SyntTax provides access to prokaryotic genomes by the means of an intuitive taxonomic tree. The selection of a particular tree node will select recursively all the child nodes up to the individual species while the parent nodes remain unselected. Node selection will also propagate correctly even if the child nodes are collapsed and therefore not visible in the interface. Displaying a taxonomic tree containing over 3000 individual rank nodes while keeping a responsive web application was not trivial. Since only portions of the tree are accessed at a given time, displaying the entire tree at once is superfluous. Taxonomic data transfer is therefore partial and only occurs upon expansion of the specific nodes. This tree loading 'on demand' was developed to avoid massive data flux from the server. In addition, to further improve performance, a significant part of the selection logic is executed locally in the web browser application itself.

Contrarily to most synteny programs using BLAST, SyntTax relies instead extensively on the Smith-Waterman-Gotoh algorithm for protein alignments. Whereas BLAST is the most widely used alignment program due to its fast execution speed, several reports pointed out the superior sensitivity of the SWG algorithm [16,17]. The choice of SWG, combined with the parallel implementation of this algorithm in SyntTax, contributes significantly to the overall performance of the web service.

### Resolution of conserved complex gene clusters

Recently, Danielou and co-workers described two particular prokaryotic syntenies with genes involved respectively in the glycogen biosynthesis/degradation pathway and in the biotin biosynthetic pathway [3]. These complex gene clusters are characterized by the presence of a number of gene permutations or inversions and by rapid sequence divergence within orthologous groups. Using SyntTax, benchmarking experiments were performed on the above syntenies as follows. Commonly used bacterial organisms were selected directly and unfamiliar lineages were retrieved by querying the taxonomy database (Figure 1B).

**Figure 3:**
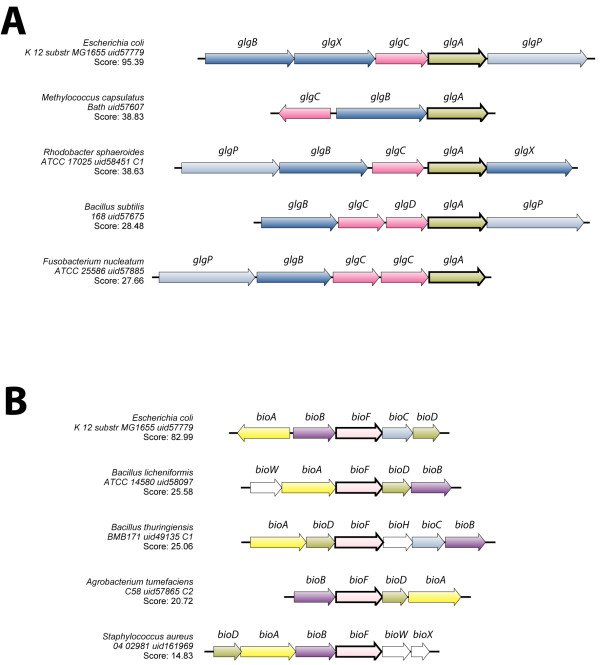
**Complex syntenies involving the *glg *and bio operon genes.** Complex syntenies were obtained by using as query sequences the *E. coli* proteins GlgA (**A**) or BioF (**B**) with the same bacterial organisms as reported by Danielou and co-workers [3]. In both cases, the characteristic high genomic plasticity of this region could be efficiently processed using SyntTax. A consistent color coding permitted the correct identification of both orthologs and paralogs. The genes corresponding to the query proteins are drawn in bold. These gene maps were produced by SyntTax in '.pdf' format and were edited as follows: the genes were renamed consistently and genes outside the synteny were removed for clarity.

In the first experiment, the GlgA protein sequence of *Escherichia coli* was extracted using the BAGET web service [12] and entered as query sequence. The results indicate that SyntTax resolved this synteny successfully (Figure 3A). Even if the diagram generated by SyntTax resembled closely the one obtained using the OTFQ algorithm [3], it is worth noting a few salient differences. Contrarily to OTFQ, SyntTax was capable of connecting all the GlgA proteins in the dataset (BLAST e-value ≤8e-69). Furthermore, the normalized BLAST score obtained in each case was very close or exceeded the recommended discriminant value of 30 used to detect *bona fide* orthology [9]. Similarly, the GlgB and GlgX genes encoding respectively the glycogen branching and the glycogen debranching enzymes in the *Gammaproteobacteria E. coli* and in the *Alphaproteobacteria Methylococcus capsulatus* were identified as orthologs since they share detectable sequence similarity (BLAST e-value ≤1e-11). SyntTax was also able to assign *B. subtilis* GlgD to the same group as GlgC (BLAST e-value ≤5e-14) whereas it remained unassigned using OTFQ. Finally, it should be mentioned that this gene arrangement is not always conserved in organisms closely related to those presented in Figure 3A. Among the 53 sequenced members of the *Bacillus* genus, it was possible to assert using SyntTax that the *B. coagulans glgP* gene is not syntenic and more importantly that the glycogen biosynthesis/degradation synton is absent in all sequenced *B. amyloliquefaciens*, *B. cellulosilyticus*, *B. clausii* and *B. pumilus* species (data not shown). The enzymes involved in this pathway are not essential in common growth conditions [18] and this might explain the absence of the corresponding gene cluster in several *Bacilli*.

The second reported complex gene cluster involves genes implicated in the biotin biosynthetic pathway [3]. Using the *E. coli* BioF protein sequence, SyntTax generated the genomic contexts shown in Figure 3B. Once again, a fully resolved synteny was obtained, connecting all relevant genes in the selected organisms. Contrarily to OTFQ, SyntTax was able to assign the *Staphylococcus aureus bioD* gene to the correct group of orthologs (BLAST e-value ≤4e-11) and to link correctly the *bioC* genes of *E. coli* and *B. thuringiensis* (BLAST e-value ≤7e-22). The rapid sequence divergence within these orthologous gene groups did not constitute an obstacle for the resolution of the corresponding complex gene clusters by SyntTax. The use of the SWG protein alignment algorithm in this web service might explain its superior sensitivity as discussed previously.

### Tracking the evolution of gene fusions to predict the existence multiprotein complexes

The detection and the resolution of complex gene clusters, described above, are not the only functions of SyntTax. This section will show that the algorithm of this web service can be used successfully to produce a more robust prediction of multiple protein-protein interactions. Most tools limit their synteny analysis to the relative positioning of clustered genes. In SyntTax, the concept of synteny is pushed one step further. This web service provide a more direct demonstration of proteins interacting with one another by allowing the visualization of gene fusions in the syntenies. Marcotte *et al.* reported over a decade ago that proteins are likely to interact when their homologs are found fused together [19]. The high proportion of false positives predicted in this way can be substantially reduced by considering only orthologs [20]. On the basis of these principles, SyntTax provides a refined and systematic approach for the study of the proteins that have evolved by modular assembly of independent domains. In addition to the exhaustive approach provided by its taxonomic capabilities, a second property of SyntTax is instrumental for this type of investigation. The synteny maps produced by this web service are drawn to the same exact scale, on the basis of GenBank annotations provided by the NCBI. This particular feature, which is absent in other resources such as GeConT2 [4], GCView [6] or OTFQ [3], allows the immediate observation of the evolution of gene size and the detection of protein fusions.

To illustrate this concept, SyntTax was used to investigate new potential partners of the highly conserved YgjD/Kae1 protein. This protein, often erroneously annotated as a protease [21], is part of a multiprotein complex involved in the biosynthesis of N6-threonylcarbamoyl adenosine (t^6^A), a universal modification found at position 37 of tRNAs decoding ANN codons [22,23]. This essential protein is present in the three domains of life and deletion mutants are characterized phenotypically by severe chromosomal loss [24]. In eukaryotes, Kae1 interacts at the biochemical and structural levels with Bud32, Pcc1, and Cgi121 in order to constitute the KEOPS/EKC complex [25]. In bacteria, however, most of these additional factors are absent and the Kae1 ortholog, named YgjD, interacts directly with the YjeE ATPase and YeaZ to constitute the YgjD complex [26]. The tRNA modification reaction was recently reconstituted *in vitro* with the addition of a fourth partner, YrdC (now called RimN) [15] even if no direct protein-protein interaction was reported between RimN and the YgjD complex. Interestingly, the archaeal organisms inherited instead the eukaryotic KEOPS/EKC complex [27,28]. The precise role of each eukaryotic or prokaryotic protein partner in the tRNA modification pathway is still unknown.

**Figure 4:**
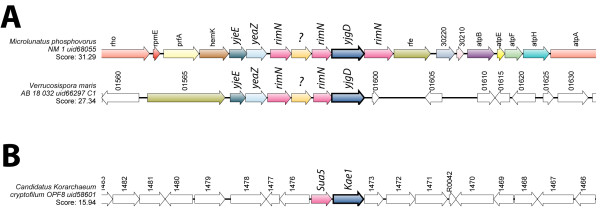
**YgjD and Kae1 syntenies involving *rimN *(*Sua5*) in bacteria and archaea.** (**A**) Complete syntenies involving the genes encoding the four subunits YgjD, YeaZ, YjeE and RimN are present in rare bacteria such as *Microlunatus phosphovorus* and *Verrcosispora maris*. A conserved unkown gene marked as '?' in present in both genomes. (**B**) A single archaea, *Korarchaeum cryptofilum* possesses Sua5 and Kae1 as neighboring genes, essentially in the same configuration as the bacteria shown in panel A.

In bacteria, gene clusters comprising *ygjD*, *yjeE*, *yeaZ* and *rimN* encoding the whole YgjD complex are too seldom to be readily observed by systematic synteny analysis. They were nevertheless detected with a C# script in which each sequenced chromosome in the database was scored for a TBLASTN inter-hit distance <8000bp using as query the protein sequence of the four subunits simultaneously. A complete synteny combining *ygjD*, *yeaZ*, *yjeE* and *rimN* was found in two organisms, *Verrucosispora maris* and *Microlunatus phosphovorus* and is illustrated using SyntTax (Figure 4A). These taxons contain respectively two or three *rimN* paralogs, and the high degree of conservation of this particular gene cluster contrasts with the variability of the neighboring regions. This situation points presumably to an horizontal gene transfer event that occurred between the ancestors of these particular species. Even if limited to two individual bacteria, this synteny argues strongly in favor of a functional interaction between these four subunits as demonstrated recently using a completely different biochemical approach [15]. Interestingly, this synteny based on bacterial genes can be extended to the archaeal domain of life. Indeed, a comparable clustering between the *ygjD* ortholog (*Kae1*) and *rimN* ortholog (*Sua5*) can be observed in the archaea *Korarchaeum cryptofilum* (Figure 4B). The same requirement of RimN/Sua5 observed in yeast t^6^A formation [29] can be therefore be predicted for the Archaeal KEOPS/EKC complex as well.

**Figure 5:**
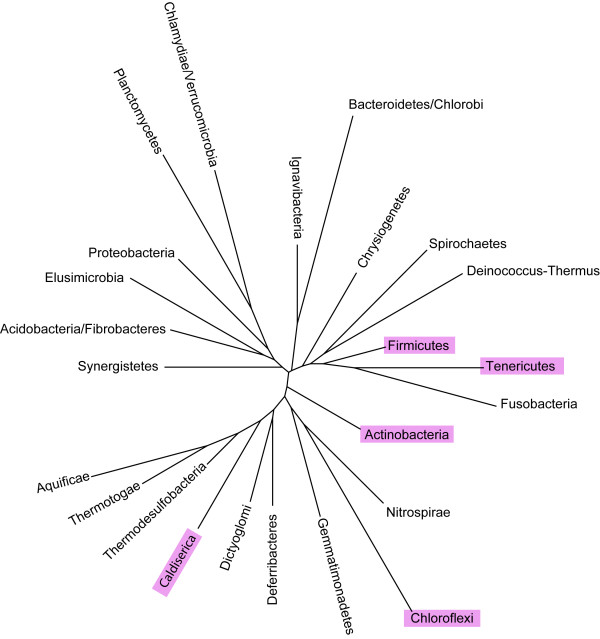
**Phylogeny of the YgjD complex.** The phylogenetic tree of the bacterial phyla based on 16S RNA sequences was obtained using a PhyML implementation [32]. Pink labels refer to phyla where several genes encoding the YgjD complex subunits are either neighboring or fused.

**Figure 6:**
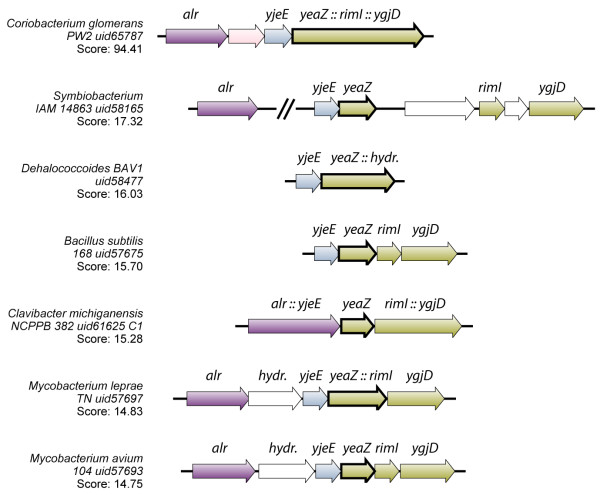
**Synteny of the genes encoding the YgjD complex.** The first 200 aminoacids encoded by the largest *yeaZ::rimI::ygjD* fusion encoded by *Coriobacterium glomerans* was used to generate the synteny of the genes encoding the YgjD complex. The organisms were chosen according to the distribution of the corresponding genes, either neighboring (*Symbiobacterium thermophilum*), adjacent (*Bacillus subtilis, Mycobacterium avium*) and fused (*Coriobacterium glomerans, Dehalococcoides BAV1, Clavibacter michiganensis, Mycobacterium leprae*). The *alr* gene is directly associated to most of these gene contexts; in the case of *S. thermophilum*, ~7kb separate *alr* from *yeaZ*.

Syntenies involving the genes encoding YgjD, YeaZ and YjeE subunits are frequently found in bacteria. SyntTax was used to produce a systematic syntenic analysis across all sequenced bacteria as follows. In a first step, genomic contexts were analyzed with SyntTax at the individual phylum level using the *E. coli* YgjD protein sequence as bait. The syntenies involving *ygjD*, *yeaZ* and *yjeE* were scored visually in the SyntTax result page. On the basis on the particular distribution of these genes, the phyla were then ranked in three categories: dispersed, co-expressed (operons) or fused. The results show that the majority of the bacterial phyla are characterized by dispersed genes encoding the YgjD complex (Figure 5). Interestingly, syntenies involving co-expressed or fused genes are restricted to gram-positive bacteria. In a second step, typical members of all the possible gene configurations were selected and analyzed in a single synteny using SyntTax. The results are summarized in Figure 6 where the various *yjeE*, *yeaZ* and *ygjD* gene clusters are shown in their respective co-expressed configuration (*Symbiobacterium, Bacillus subtilis, Mycobacterium avium*) and fused configurations (*Coriobacterium glomerans, Dehalococcoides, Clavibacter michiganensis, Mycobacterium leprae*). Several additional observations could be made. First, a number of alternative gene fusions significantly associate both YeaZ and YgjD to another subunit, RimI which is responsible of the N^α^-acetylation of ribosomal protein S18 [30]. A triple fusion associating these three factors is particularly noticeable in the *C. glomerans* taxon. A second observation indicates that the *alr* gene, encoding the constitutive alanine racemase, is also strongly associated with this gene cluster, either as an individual gene or fused to *yjeE* in *C. michiganensis*. Finally, an hydrolase gene is found immediately upstream *yjeE* in *M. leprae* and *M. avium* and a putative hydrolase (albeit unrelated) is fused to YeaZ in *Dehalococcoides*. SyntTax correlates the presence of *ygjD* with that of three new genes encoding the acetyl transferase RimI, the alanine racemase Alr and an ubiquitous hydrolase. Among these three new predicted YgjD-interacting factors, only RimI could be detected using the STRING web service [31]. Biochemical data have shown that the bacterial YgjD, YeaZ, YjeE and RimN interact and are involved in t^6^A tRNA modification, a process ensuring optimal translation of ANN codons [22]. The results of the present work, obtained with a completely different genomic approach, confirm the functional link between these four functions. In addition, these syntenies strongly suggest a further association of the YgjD complex with RimI and Alr proteins.

## Conclusions

In this post-genomic era, the accumulation of completely sequenced genomes provides, in theory, the opportunity to assign a biological role to a growing number of proteins. However, newly sequenced prokaryotic organisms not only introduce a considerable amount of additional orphan proteins but also a certain degree of redundancy. The scientific community is therefore expected to produce increasing efforts in order to keep a favorable proportion of proteins with known functions over the total amount of protein sequences. Only time consuming wet bench experimentation is able to produce the ultimate proof of functions and it constitutes the bottleneck in this process. Fortunately, comparative genome analysis assists the biologist in this endeavor by prioritizing targets for experimental study [33]. The development of new bioinformatics tools that can adapt to continuously expanding genomic datasets is therefore fundamental. The present work has focused on the development of SyntTax, a new synteny web service whose predictive robustness increases with the amount of available prokaryotic genomes. This property is due to the taxonomic capabilities offered by this web tool and which is not available in comparable implementations.

The results presented here show that the quality of complex syntenies produced in real time by SyntTax equals or exceeds those obtained by other methodologies such as OTFQ which requires hours of systematic calculation on a finite set of genomes. The extensive use of the SWG protein alignment algorithm contributes positively to its performance and sensitivity. Several unique additional SyntTax properties were instrumental for the analysis of the *ygjD* gene cluster. Beyond the confirmation of known interacting factors in this complex, SyntTax was able to predict the interplay between new potential partners composing this multiprotein machinery involved in the bacterial tRNA modification.

The SyntTax web server is built upon the proven Absynte synteny algorithm and allows full access to complete taxonomic records for each sequenced archaea and bacteria in the NCBI database. This tool is capable of adapting rapidly to the sequencing of new genomes due to the daily automated update of its databases. The taxonomic capabilities of this web service are not available in other tools and add a new dimension to synteny analysis by providing robust results while reducing computing time. Exhaustive access to prokaryotic lineages is not available in similar tools such as GeCont2, PSAT or GCVIEW. Although solely based on genomic sequences, annotations and taxonomic data, the accurate illustration of genomic contexts produced by SyntTax is able to provide exhaustive cutting edge predictive information on protein-protein interaction. These predictions complement adequately those provided by other tools such as STRING which uses a complex combination of genomic, post-genomic and text databases. The principles shown here demonstrated that the high number of sequenced genomes and their increasing redundancy no longer constitute a burden. This wealth of information can indeed be used effectively to target specific aspects of the evolution of prokaryotic gene clusters and to yield insights on the molecular interaction between the corresponding proteins. Predictive models, such as those produced by SyntTax, would constitute interesting challenges for wet bench experimentalists.

## Availability and requirements

Project name: SyntTax

Project home page: http://archaea.u-psud.fr/synttax.

Operating system(s): platform independent.

Programming language: C#, ASP.NET and Javascript.

Other requirements: internet connection.

License: none required.

Any restrictions to use by non-academics: no restriction.

## Competing interests

The author declares that he has no competing interests.
